# Association between the c.*229C>T polymorphism of the topoisomerase IIβ binding protein 1 (*TopBP1*) gene and breast cancer

**DOI:** 10.1007/s11033-012-2424-z

**Published:** 2013-01-01

**Authors:** Ewa Forma, Ewa Brzeziańska, Anna Krześlak, Grażyna Chwatko, Paweł Jóźwiak, Agnieszka Szymczyk, Beata Smolarz, Hanna Romanowicz-Makowska, Waldemar Różański, Magdalena Bryś

**Affiliations:** 1 Department of Cytobiochemistry, University of Łódź, Pomorska 141/143, 90-236 Lodz, Poland; 2 Department of Molecular Bases of Medicine, Medical University of Łódź, Pomorska 251, 92-213 Lodz, Poland; 3 Department of Environmental Chemistry, University of Łódź, Pomorska 163, 90-236 Lodz, Poland; 4 Department of Clinical Pathomorphology, Polish Mother’s Memorial Hospital Research Institute, Łódź, Rzgowska 281/289, 93-338 Lodz, Poland; 5 2nd Department of Urology, Medical University of Łódź, Pabianicka 62, 93-513 Lodz, Poland

**Keywords:** Topoisomerase IIβ binding protein 1, Polymorphism, Genetic variation, Breast cancer

## Abstract

Topoisomerase IIβ binding protein 1 (TopBP1) is involved in cell survival, DNA replication, DNA damage repair and cell cycle checkpoint control. The biological function of TopBP1 and its close relation with BRCA1 prompted us to investigate whether alterations in the *TopBP1* gene can influence the risk of breast cancer. The aim of this study was to examine the association between five polymorphisms (rs185903567, rs116645643, rs115160714, rs116195487, and rs112843513) located in the 3′UTR region of the *TopBP1* gene and breast cancer risk as well as allele-specific gene expression. Five hundred thirty-four breast cancer patients and 556 population controls were genotyped for these SNPs. Allele-specific TopBP1 mRNA and protein expressions were determined by using real time PCR and western blotting methods, respectively. Only one SNP (rs115160714) showed an association with breast cancer. Compared to homozygous common allele carriers, heterozygous and homozygous for the T variant had significantly increased risk of breast cancer (adjusted odds ratio = 3.81, 95 % confidence interval: 1.63–8.34, *p* = 0.001). Mean TopBP1 mRNA and protein expression were higher in the individuals with the CT or TT genotype. There was a significant association between the rs115160714 and tumor grade and stage. Most carriers of minor allele had a high grade (G3) tumors classified as T2-T4N1M0. Our study raises a possibility that a genetic variation of TopBP1 may be implicated in the etiology of breast cancer.

## Introduction

Breast cancer is the most frequently diagnosed cancer and one of the leading causes of cancer death among women worldwide [1]. In Poland breast cancer is the second most common cause of cancer death in women. Moreover, breast cancer incidence rates have been reported to increase by up to 5 % per year in developing countries [2]. Although environmental factors and lifestyle could contribute to the increased breast cancer risk, genetic factors are also implicated in the pathogenesis of the disease. Recent genome-wide and candidate gene association studies have identified some low-penetrance variants associated with breast cancer [3, 4]. However, despite great progress in the breast cancer studies the molecular mechanisms that contribute to breast carcinogenesis remain poorly understood. Thus, there is a necessity to identify all breast cancer susceptibility genes.

TopBP1 protein was first identified as an interacting partner for topoisomerase IIβ. *TopBP1* gene comprising 28 exons is located on chromosome 3q22.1 and encodes a 1,522 amino acid protein (180 kDa) [5]. TopBP1 protein seems to be essential for maintenance of chromosomal integrity and cell proliferation. This protein appears to be involved in DNA replication, DNA damage response and cell cycle checkpoint control [6, 7]. The most striking feature of TopBP1 is that it has eight BRCA1 C-terminal (BRCT) domains which were first identified in breast cancer gene 1 (BRCA1) [8, 9]. BRCT domains, about 90 amino acids in length are hydrophobic and are involved in interaction with other proteins as well as in interaction with single and double-stranded DNA [10]. The C-terminal region of TopBP1 containing two BRCTs is responsible for interaction with topoisomerase and shows considerable similarities with BRCA1. Apart from structural similarities TopBP1 shares many other common features with BRCA1. Both TopBP1 and BRCA1 are strongly induced during S phase. Following ionizing radiation, TopBP1 is recruited to DNA breaks and colocalizes with Nbs1 (Nijmegen breakage syndrome 1), BRCA1 and 53BP1 (p53-binding protein 1) in nuclear foci. TopBP1 and BRCA1 also colocalize with proliferating cell nuclear antigen at stalled replication forks after a replication block. Both proteins are phosphorylated by ATM (ataxia telangiectasia mutated) kinase in response to DNA damage and DNA replication stress [5]. Moreover, it was shown that TopBP1 is involved in regulation of p53 activities during normal growth.

The biological function of TopBP1 and its close relation with BRCA1 prompted us to investigate whether genetic alterations in the *TopBP1* gene can influence the risk of breast cancer. In present study we tested the effect of SNPs potentially located in the 3′UTR (3′untranslated region) region of the *TopBP1* gene and listed in NCBI’s (National Center for Biotechnology Information) SNP database on breast cancer risk as well as on allele-specific mRNA/protein expression. There are five such SNPs—rs185903567 (G/A), rs116645643 (A/G), rs115160714 (C/T), rs116195487 (C/G), and rs112843513 (C/delC)]. We correlated obtained results with clinicopathological characteristics.

## Materials and methods

### Study population

This study involved 534 women with non-hereditary infiltrating ductal breast carcinomas (age range 43–81, mean age 54.76 ± 7.35) recruited between May 2003 and November 2010. The patients had a confirmed diagnosis of ductal breast cancer based on histopathological evaluation and were under treatment at the Polish Mothers Memorial Hospital Research Institute, Łódź, Poland. None of the recruited patients received preoperative chemo- or radiotherapy. Patients diagnosed with previous breast tumors or with tumors located elsewhere were excluded.

A group of 556 healthy Polish individuals were collected from the hospital routine controls of health and used as control. They were non-related women, that have never been diagnosed with breast tumors, other tumors or chronic disease and were randomly selected and frequency matched to the cases on age (age range 34–83, mean age 51.27 ± 11.18).

Blood samples were collected from all women participating in the study and additionally breast cancer tissues were obtained from patients with breast neoplasms. We enrolled only women born and living in central Poland (Łódź region). Informed consent was obtained from patients and controls, and ethics approval was obtained from the ethics commission of the Polish Mother’s Memorial Hospital, Research Institute (G4/2011).

### Lifestyle risk factors

Study participants were interviewed using questionnaire that included socio-demographic, medical history, health related information, alcohol intake, smoking status, menstrual and reproductive histories, and exogenous hormone use. Medical records of patients were thoroughly reviewed. The tumor stages were classified according to the 1997 TNM staging system of the American Joint Committee on Cancer [11]. Tumors were graded according to the Bloom and Richardson classification, modified by Elston and Ellis [12]. A positive family history of breast cancer was defined as reporting of breast cancer in one or more first degree relatives. Body mass index (BMI) was calculated based on current weight in kilograms divided by height in meters squared.

The subjects were classified as never/rare drinkers, ex-drinkers, or current drinkers who consumed 1–8.9 U/week (light drinkers), 9–17.9 U/week (moderate drinkers), or 18 U/week (heavy drinkers), where 1 U = 22 g ethanol [13].

According to smoking status patients and controls were grouped into ‘‘never’’, “former” and “current” based on self-reported usage. Participants who reported smoking at least 100 cigarettes in their lifetime and who, at the time of survey, smoked either every day or some days were defined as Current Smoker. Participants who reported smoking at least 100 cigarettes in their lifetime and who, at the time of the survey, did not smoke at all were defined as Former Smoker. Participants who reported never having smoked 100 cigarettes were defined as Never Smoker.

Menopause was defined as the time of the last menstrual period (or menstrual flow of any amount). None of the women involved in the study had undergone a hysterectomy. Regular drug usage was defined as self-report use of oral contraceptives or menopausal hormones for 6 months or longer.

### Blood sample collection, SNP selection and genotyping

Each genomic DNA sample was extracted from peripheral blood using FlexiGene^®^ DNA Kit (Qiagen GmbH, Hilden, Germany). DNA concentration was determined by spectrophotometry. The single nucleotide polymorphisms (SNPs) rs185903567 (G/A), rs116645643 (A/G), rs115160714 (C/T), rs116195487 (C/G), rs112843513 (C/delC) located at the 3′UTR of *TopBP1* gene and listed in the NCBI’s SNP database were evaluated. These polymorphisms were analyzed by PCR amplification and direct sequencing. The amplified region included the entire 3′UTR region (nucleotides 4629-5289; NCBI Reference Sequence: NM_007027.3). Briefly, amplification was carried out in a final volume of 25 μl containing 100 ng genomic DNA, 0.3 μM of forward (5′-TGGGACTGGATTATCACAAAAG-3′) and reverse (5′-CTTTTATTCTTTATTGTCACATTTTCC-3′) primers, 0.2 mM dNTPs (deoxyribonucleoside triphosphates), 2 mM MgCl_2_, 1 × Buffer (Applied Biosystems, Darmstadt, Germany) and 1 U AmpliTaq Gold (Applied Biosystems, Darmstadt, Germany). PCR conditions were 94 °C for 5 min; 35 cycles with denaturation at 94 °C for 30 s, annealing at 61 °C for 30 s, and elongation at 72 °C for 30 s; and a final extension at 72 °C for 10 min. Purified PCR products were then sequenced using BigDye^®^ Terminator v3.1 Cycle Sequencing Kit (Applied Biosystems, Darmstadt, Germany) and electrophoresed on a 3730 DNA Analyzer (Applied Biosystems, Foster City, CA).

### Total RNA extraction and cDNA synthesis

Total RNA was extracted from breast cancer tissues using TRI Reagent^®^ (Sigma Aldrich Corp. St. Louis, MO, USA) according to manufacturer’s protocol. RNA was eluted in 20 μl RNase-free water, quantified by spectrophotometry at 260 nm and stored at −20 °C. RNA with a 260/280 nm ratio in range 1.8–2.0 was considered high quality. First-strand cDNAs were obtained by reverse transcription of 1 μg of total RNA using RevertAid™ First Strand cDNA Synthesis Kit (Fermentas UAB, Vilnius, Lithuania) following the manufacturer’s protocol.

### Real time quantitative PCR

For real-time PCR analysis of *TopBP1* mRNA in normal and pathological tissues, TaqMan^®^ Gene Expression Assays (Applied Biosystems, Bedford, MA, USA) were used according to the manufacturer’s instruction. Before starting the real-time PCR analysis we used the NormFinder algorithm to select the best reference gene (http://www.mdl.dk). We chose *GAPDH* (glyceraldehyde 3-phosphate dehydrogenase) gene because it had the lowest stability value−0.017. The fluorogenic, FAM labeled probes and the sequence specific primers for *TopBP1* and *GAPDH* were obtained as inventoried assays Hs00199775_m1 and Hs99999905_m1, respectively (Applied Biosystems, Bedford, MA, USA). The reactions were performed in duplicate. A positive result was defined by a threshold cycle (Ct) value lower than 40 (the Ct value is determined by the number of cycles needed to exceed the background signal). Ct value of all positive results were lower than 30. Abundance of *TopBP1* mRNA in studied material was quantified by the ΔCt method. ΔCt (Ct_*TopBP1* _− Ct_*GAPDH*_) values were recalculated into relative copy number values (number of copies of *TopBP1* mRNA per 1,000 copies of *GAPDH* mRNA).

### Western blotting analysis

Tissue homogenate was obtained from each sample in the presence of the serine protease inhibitor PMSF (phenylmethylsulfonyl fluoride) and 10 mM sodium molybdate. The protein content was estimated by modified Lowry method using bovine serum albumin as standard. Homogenate proteins (50 μg protein/lane) were resolved by 8 % SDS-PAGE and electroblotted onto Immobilon-P transfer membranes (Millipore, Bedford, MA, USA). The blots were incubated 1 h with rabbit polyclonal anti-TopBP1 (Abcam, Cambridge, UK) in a 1:1,000 dilution. After being washed three times with TBST (Tris buffered saline with Tween-20), the membranes were incubated 1 h with goat anti-rabbit antibodies conjugated with horseradish peroxidase (1:5,000 dilution). The membranes were again washed three times with TBST and incubated with peroxidase substrate solution (3,3′-diaminobenzidine—DAB). Gel-Pro^®^ Analyzer software (Media Cybernetics Inc., Bethesda, MD, USA) was used for densitometry analysis of protein bands. The integrated optical density (IOD) of the bands, in a digitized picture, was measured.

### Evaluation of estrogen receptor and progesterone receptor

Estrogen receptor (ER) and progesterone receptor (PR) status was determined by immunohistochemical method as part of the routine clinical practice. Using the immunohistochemical assay, tumors were classified as positive if more than 10 % of the cells showed nuclear staining for the receptor. This information was received together with the characteristics of clinical material.

### Quality control

For quality control purposes, 10 % of samples were randomly selected, and sequence analysis performed, with 100 % concordance to the genotype. Laboratory personnel were unable to distinguish among case, control, and quality control samples.

### Statistical data analysis

Genotype distributions were evaluated for agreement with Hardy–Weinberg equilibrium by the Chi-square test. Unconditional multiple logistic regression models were used to calculate odds ratios (ORs) and 95 % confidence intervals (CIs) for the association of genotype with breast cancer risk. Genotype data were analyzed with the homozygote of the common allele as the reference group. Variants of homozygotes and heterozygotes were combined to evaluate the dominant effect. For each SNP, trend tests were conducted by assigning the ordinal values 1, 2, and 3 to homozygous wild-type, heterozygous, and homozygous variant genotypes, respectively, and by adding these scores as a continuous variable in logistic regression model. All multivariate models were adjusted for age, family history, obesity, smoking status, parity, menopausal status, and use of contraceptive and menopausal hormones. Since levels of *TopBP1* mRNA and protein expression in studied material specimens did not show normal distribution (Kolmogorov–Smirnov test) the non-parametrical statistical tests (Mann–Whitney *U* test, Kruskal–Wallis test with post hoc multiple comparisons, Chi square test or the Spearman rank correlation test) were applied. Reported *p* values were two-sided. Probabilities were considered significant whenever p-value was lower than 0.05. All analyses were completed using SAS software (version 9.0 SAS Institute, Cary, NC, USA).

## Results

### Characteristics of subjects

The distributions of sociodemographic characteristics, lifestyle risk factors and clinical characteristics of the patients are shown in Tables 1 and 2, respectively. All patients and healthy subjects were Caucasian. The cases were slightly older (mean age 54.76 ± 7.35 vs. 51.27 ± 11.18), were more likely to have an BMI equal or greater than 30 (39.9 vs. 28.9 %) and more likely to use contraceptives (estrogens and progestins) and menopausal hormones 64.4 vs. 50.1 % and 42.3 vs. 30.9 %, respectively, than controls. Moreover, both groups slightly differ in smoking status. More patients currently smoke (37.1 vs. 26.2 %) and fewer had never smoked (18.0 vs. 27.9 %,) than women in control group.

**Table 1:** Selected baseline characteristics of breast cancer cases and controls with questionnaire data

	Cases (*n*, %) (*n* = 534)	Controls (*n*, %) (*n* = 556)	*p* ^a^
Age (years)			
<45	123 (23.0)	189 (34.0)	
45–54	133 (25.0)	139 (25.0)	
55–64	150 (28.1)	122 (21.9)	
>64	128 (23.9)	106 (19.1)	<0.001
Family history of breast cancer^b^			
Yes	64 (11.9)	50 (9.0)	
No	470 (88.1)	506 (91.0)	0.11
Obesity (BMI ≥30 kg/m^2^)^c^			
Yes	213 (39.9)	161 (28.9)	
No	321 (60.1)	395 (71.1)	<0.0001
Smoking status^d^			
Never smokers	96 (18.0)	155 (27.9)	
Formet smokers	240 (44.9)	255 (45.9)	
Current smokers	198 (37.1)	146 (26.2)	<0.001
Alcohol intake^e^			
Never/rare	43	27	
Light	201	223	
Moderate	163	171	
Heavy	116	127	
Ex-drinker	11	8	0.24
Menarche (years)			
10	11 (2.1)	0 (0.0)	
11	101 (18.9)	106 (19.2)	
12	171 (32.1)	200 (35.9)	
13	144 (26.9)	167 (30.0)	
14	91 (17.1)	72 (12.9)	
≥15	16 (2.9)	11 (2.0)	<0.01
Used oral contraceptives^f^			
Yes	344 (64.4)	283 (50.1)	
No	190 (35.6)	273 (49.9)	<0.0001
Parity			
Nulliparous	114 (21.3)	128 (23.0)	
1	125 (23.4)	144 (25.9)	
2	140 (26.2)	156 (28.0)	
3	98 (18.3)	94 (16.9)	
≥4	57 (10.8)	34 (6.2)	0.07
Menopausal status^g^			
Premenopausal	192 (35.9)	228 (41.0)	
Postmenopausal	342 (64.1)	328 (59.0)	0.09
Use of menopausal hormones^f^			
Never	308 (57.7)	384 (69.1)	
Estrogen	144 (27.0)	94 (16.9)	
Progestin	32 (6.0)	23 (4.1)	
Combined	50 (9.3)	55 (9.9)	<0.001

^a^χ^2^ test

^b^Family history defined as self-reporting of at least one first-degree relative with known breast cancer

^c^Body mass index (BMI) was calculated as current weight in kilograms divided by height in meters squared

^d^Participants who reported smoking at least 100 cigarettes in their lifetime and who, at the time of survey, smoked either every day or some days were defined as current smoker. Participants who reported smoking at least 100 cigarettes in their lifetime and who, at the time of the survey, did not smoke at all were defined as former smoker. Participants who reported never having smoked 100 cigarettes were defined as never smoker

^e^Never/rare, <1 U/week; light, 1–8.9 U/week; moderate, 9–17.9 U/week; heavy, ≥18 U/week; where 1 U = 22 g ethanol

^f^Regular drug use was defined as self-report use of oral contraceptives for 6 months or longer

^g^Menopause was defined as the time of the last menstrual period (or menstrual flow of any amount). None of the women involved in the study had undergone a hysterectomy

**Table 2:** The clinicopathological characteristics of 534 patients with breast cancer

Variable	Mean ± SD or *n* (%)
Age (years)	54.76 ± 7.35
Histopathological grading	
1	112 (21.0)
2	208 (38.9)
3	128 (24.0)
1 + 2	86 (16.1)
Primary tumor stage	
T1-2N0M0	389 (72.8)
T2-4N1M0	145 (27.2)
Tumor size	
≤2 cm	313 (58.6)
2–5 cm	219 (41.0)
>5 cm	2 (0.4)
ER and PR status	
ER+PR+	341 (63.8)
ER+PR−/ER−PR+	112 (21.0)
ER−PR−	81 (15.2)

### Genotypes and genotypic distribution in patients and control subjects

Genotype distributions for TopBP1 polymorphisms in 534 breast cancer patients and 556 control subjects are summarized in Table 3. Two SNPs (rs116195487 and rs185903567) were not observed in the 3′UTR region of *TopBP1* gene in our studied groups. During the study, we have not identified any new mutations, not listed in the SNP databases. All cases and controls were common allele carriers. Only one SNP (rs115160714) showed an association with breast cancer. The frequency of individuals who carried (T) allele was significantly higher in cases group (3.1 %) than in controls group (0.8 %; *p* < 0.001). Compared to homozygous common allele carriers, heterozygous for the T variant were found to be at a significant 3.54-fold increased risk of breast cancer (95 % CI = 1.56–8.39; *p* = 0.002). The TT genotype even more increased breast cancer risk compared with those harboring the CC genotypes (OR = 5.40, 95 % CI = 0.63–46.64; p = 0.004). The comparison of combined genotypes is shown in Table 4. Most cases and controls showed only one SNP polymorphism. However, nineteen cases (3.5 %) were heterozygotes for rs115160714 and had a C deletion in rs112843513.

**Table 3:** Frequency distribution of the *TopBP1* genotypes/alleles in cases and controls, and the risk of breast cancer

Variables	Cases (*n*, %)/controls (*n*, %)	OR (95 % CI)^a^	*p*
rs116645643			
AA	512 (95.9)/545 (98.0)	1.00 (ref.)	
AG	21 (3.9)/11 (2.0)	2.19 (0.96–4.31)	>0.05
GG	1 (0.2)/0 (0.0)	–	
A	1045 (97.8)/1101 (99.0)	1.00 (ref.)	
G	23 (2.1)/11 (0.1)	2.22 (1.11–4.52)	0.03
p-trend^b^		0.05	
AG or GG vs. AA^c^		2.16 (1.02–4.47)	>0.05
AG or AA vs GG^d^		–	–
rs115160714			
CC	506 (94.7)/548 (98.6)	1.00 (ref.)	
CT	23 (4.4)/7 (1.2)	3.54 (1.56–8.39)	0.002
TT	5 (0.9)/1 (0.2)	5.40 (0.63–46.64)	0.004
C	1035 (96.9)/1103 (99.2)	1.00 (ref.)	
T	33 (3.1)/9 (0.8)	3.97 (1.81–8.25)	0.001
p-trend^b^		0.0006	
CT or TT vs. CC^c^		3.81 (1.63–8.34)	0.001
CT or CC vs. TT^d^		5.23 (0.65–45.07)	0.15
rs112843513			
CC	391 (73.2)/389 (70.0)	1.00 (ref.)	
C/delC	143 (26.8)/167 (30.0)	0.80 (0.67–1.06)	0.28
delC/delC	0 (0.0)/0 (0.0)	–	
C	925 (86.6)/945 (85.0)	1.00 (ref.)	
delC	143 (13.4)/167 (15.0)	0.83 (0.62–1.14)	0.22
p-trend^b^		0.24	
C/delC or delC/delC vs. CC^c^		0.86 (0.65–1.11)	0.23
C/delC or CC vs. delC/delC^d^		–	–

*del* allele deletion

^a^Adjusted for age, family history, smoking status, alcohol intake, BMI, menarche, parity, menopausal status, and use of contraceptive and menopausal hormones

^b^Testing additive genetic model (Cochran–Armitage test for trend)

^c^Testing dominant genetic model

^d^Testing recessive genetic model

**Table 4:** The distribution of *TopBP1* polymorphisms combined genotypes in breast cancer cases and controls

	rs115160714		
	CC	CT	TT
rs116645643 AA and rs112843513 CC	371 (69.5)/379 (68.2)	1 (0.2)/1 (0.2)	0 (0.0)/1 (0.2)
rs116645643 AA and rs112843513 C/delC	117 (21.9)/160 (28.8)	19 (3.5)/4 (0.7)	4 (0.7)/0 (0.0)
rs116645643 AG and rs112843513 CC	17 (3.2)/7 (1.2)	1 (0.2)/1 (0.2)	0 (0.0)/0(0.0)
rs116645643 AG and rs112843513 C/delC	0 (0.0)/0 (0.0)	2 (0.4)/1 (0.2)	1 (0.2)/0 (0.0)
rs116645643 GG and rs112843513 CC	1 (0.2)/2 (0.3)	0 (0.0)/0 (0.0)	0 (0.0)/0 (0.0)

The table shows the number of cases and the percentage of genotype occurrence, respectively, in the study group and control population

### Association of rs115160714 with clinical and environmental parameters

Of the 534 breast cancer patients, 406 (76.0 %) had a low grade tumor (grades G1 and G2), and 128 (24.0 %) had a high grade tumor (G3). Most tumors, 389 (72.8 %) was classified as T1-2N0M0, and the remaining 145 (27.2 %) was T2-4N1M0 (Table 5). There was a significant association between CT and TT genotypes and tumor grade or stage. Most carriers of minor allele had a high grade tumors classified as T2-4N1M0 (Table 5).

**Table 5:** Adjusted odds ratio for relation between *TopBP1* genotypes and different tumor grades and stages

Variables	Grade (*n*, %)		OR (95 % CI)^a^	*p*
	Low grade	High grade		
	(*n* = 406)	(*n* = 128)		
CC	397 (97.8)	109 (85.1)	1.00 (ref.)	
CT	8 (2.0)	15 (11.7)	6.83 (2.75–16.86)	0.0001
TT	1 (0.2)	4 (3.2)	14.59 (1.56–134.81)	0.002
C	802 (98.8)	233 (91.0)	1.00 (ref.)	
T	10 (1.22)	23 (9.0).	7.94 (3.66–17.18)	0.0001

	Tumor stages (*n*, %)			
	T1-2N0M0	T2-4N1M0		
	(*n* = 389)	(*n* = 145)		
CC	382 (98.2)	124 (85.5)	1.00 (ref.)	
CT	5 (1.3)	18 (12.4)	11.07 (3.92–31.52)	0.0001
TT	2 (0.5)	3 (2.1)	4.64 (0.77–28.23)	0.07
C	769 (98.8)	266 (91.7)	1.00 (ref.)	
T	9 (1.2)	24 (8.3)	7.72 (3.46–17.03)	0.0001

^a^Adjusted for age, family history, smoking status, alcohol intake, BMI, menarche, parity, menopausal status, and use of contraceptive and menopausal hormones

The analysis of SNP polymorphism (rs115160714) in smokers and non-smokers groups showed that smoking is a significant breast cancer risk factor in case of T allele carriers (Table 6). There was no association between alcohol intake and breast cancer risk.

**Table 6:** Comparison of the *TopBP1* genotypes prevalence according to smoking status and adjusted odds ratio for relation between *TopBP1* genotypes and smoking

	Genotypes						
	Cases (*n*, %)			Controls (*n*, %)			
Smoking status	CC	CT	TT	CC	CT	TT	*p* ^a^
	*n* = 506	*n* = 23	*n* = 5	*n* = 548	*n* = 7	*n* = 1	
Smokers^b^	163 (30.5)	7 (1.3)	3 (0.6)	224 (40.3)	2 (0.3)	1 (0.2)	0.04
Non-smokers^b^	343 (64.2)	16 (3.0)	2 (0.4)	324 (58.3)	5 (0.9)	0 (0.0)	0.11

Genotypes in smokers	Cases (*n*, %)	Controls (*n*, %)	OR (95 % CI)^c^	*p*
CC	163 (94.2)	224 (98.7)	1.00 (ref.)	
CT	7 (4.0)	2 (0.9)	4.82 (0.96–23.76)	0.03
TT	3 (1.8)	1 (0.4)	4.11 (0.45–40.37)	0.19
C	333 (96.2)	450 (99.1)	1.00 (ref.)	
T	13 (3.8)	4 (0.9)	4.35 (1.43–13.66)	0.005

^a^χ^2^ test

^b^Smoking was grouped into “smokers” and “non-smokers” based on self-reported usage or data obtained from family. Smoking factor was considered positive when the subject smoked at least five cigarettes in a day for more than 1 year during the last 10 years

^c^Adjusted for age, family history, alcohol intake, BMI, menarche, parity, menopausal status, and use of contraceptive and menopausal hormones

### Association between TopBP1 genotypes and mRNA/protein expression in breast cancer tissue

**Fig. 1:**
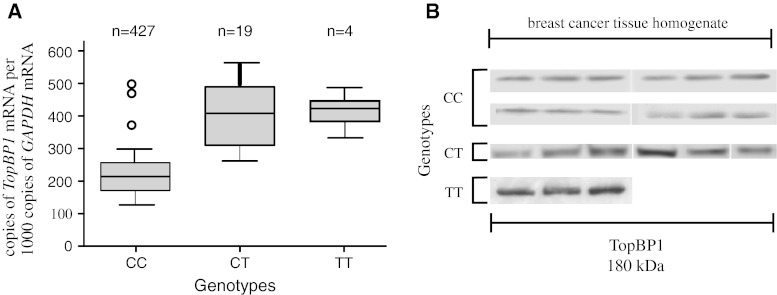
The relationship between TopBP1 mRNA and protein expression and the rs115160714 genotype in breast cancers. **a** Expression of *TopBP1* gene measured by real-time PCR in relation to genotype. **b** Western blotting analysis of TopBP1 expression measured in relation to genotype. Figure shows the representative results of TopBP1 immunodetection in breast cancer tissue homogenates (50 μg protein per lane)

We found that mean *TopBP1* mRNA expression was lower in the case of individuals with the CC genotype than in case of minor allele carriers, i.e. CT heterozygotes and TT homozygotes (223.0, 412.0 and 428.5 copies of *TopBP1* mRNA per 1000 copies of *GAPDH* mRNA, respectively, *p* < 0.05 for all comparisons) (Table 7; Fig. 1a). We found TopBP1 protein expression in 81.2, 69.5 and 60.0 % of breast tissue homogenate samples of CC, CT, and TT genotype carriers, respectively. Although the protein expression was more frequently observed in common allele carriers group, the mean expression level was lower than in minor allele carriers (84.6, 118.2, 127.4 IOD relative units, respectively, *p* < 0.05 for all comparisons) (Table 7; Fig. 1b). There was a statistically significant correlation between *TopBP1* mRNA and protein expressions (Spearman correlation coefficient for CC and CT genotype 0.76 and 0.82, respectively, *p* < 0.05 for all comparisons). However, not in all cases with positive mRNA expression we could detected TopBP1 protein. Both mRNA and protein was detected in 306 of 506 CC samples, in 14 of 23 of CT samples and 3 of 5 TT samples.

**Table 7:** Comparison of TopBP1 mRNA and protein expression in breast cancer tissues with genotypes of *TopBP1* gene

Gene			
Genotypes	Positive expression (*n*, %)	*TopBP1* mRNA expression (copies of *TopBP1* mRNA per 1,000 copies of *GAPDH* mRNA)	*p* ^a^
CC	427/506 (84.3)	223.0 ± 57.0	
CT	19/23 (82.6)	412.0 ± 138.0	0.030
TT	4/5 (80.0)	428.5 ± 112.2	0.007

Protein			
	Positive expression (*n*, %)	TopBP1 protein expression [integrated optical density (IOD) relative units] homogenate fraction	*p* ^a^
CC	411/506 (81.2)	84.6 ± 21.5	
CT	16/23 (69.5)	118.2 ± 28.5	0.041
TT	3/5 (60.0)	127.4 ± 19.3	0.025

Results are given as mean ± standard error

^a^Differences between the three groups were evaluated with Kruskal–Wallis test with post hoc multiple comparisons

## Discussion

The biological functions of TopBP1 protein as well as its close relation with BRCA1 suggest a crucial role of this protein in the maintenance of genome integrity and cell cycle regulation. Published data on the involvement of TopBP1 in breast carcinogenesis are very limited. However, the aberrant expression of TopBP1 protein in breast cancer was shown. Immunohistochemical analysis of TopBP1 level demonstrated that this protein was expressed almost exclusively in nuclei of the normal breast epithelium while in breast cancer samples TopBP1 was detected in nucleus and/or in cytoplasm [14]. Analysis of TopBP1 protein expression in feline and canine mammary neoplasms revealed that most TopBP1 immunohistochemical staining was nuclear but both nuclear and cytoplasmic staining was observed as the degree of malignancy increased. Expression of TopBP1 protein was also correlated with histological grade of neoplasms [14, 15]. Patients with overexpression of TopBP1 tend to have higher grades of breast cancer and negative estrogen receptor status compared with those without overexpression of this protein and have significantly shorter overall survival time [16]. The results of our earlier studies concerning expression of TopBP1 in hereditary breast cancer showed lower *TopBP1* mRNA expression in lobular carcinoma compared with ductal carcinoma. The level of *TopBP1* mRNA appeared to be lower in poorly differentiated (III grade) hereditary breast cancer in comparison with moderately (II grade) and well-differentiated cancer (I grade). However, the immunohistochemistry and Western blot analyzes showed significantly increased of TopBP1 protein level in poorly differentiated breast cancer (III grade). Our data suggested that increased level of TopBP1 protein might be associated with progression of hereditary breast cancer [17].

Two SNPs (rs116195487 and rs185903567) of the five listed in the NCBI’s SNP database were not observed in the 3′UTR region of *TopBP1* gene in our studied groups. This allows to conclude that these polymorphisms do not occur in the Polish population.

In this study, we demonstrated for the first time that rs115160714 in the 3′UTR region of *TopBP1* gene is significantly associated with breast cancer risk. Compared to homozygous common allele carriers, heterozygous for the T variant were found to be at a significant 3.54-fold increased risk of developing breast cancer (95 % CI = 1.56–8.39; *p* = 0.002).

Since genetic alteration to the 3′UTR sequence can increase or decrease the half- life of the mRNA leading to greater or lesser protein levels we presumed that rs115160714 may affect TopBP1 expression. We found out that mean TopBP1 mRNA and protein levels were higher in case of individuals with CT or TT genotype.

There are several regulatory sequences in the 3′UTR that can affect expression, i.e. polyadenylation signal, AU-rich elements and binding sites for miRNAs [18]. The rs115160714 was not located in or close to any of these sites except miRNAs binding sites. Thus we suggested that polymorphism rs115160714 could change stability of *TopBP1* mRNA by affecting miRNA binding.

MicroRNAs are a class of regulatory RNAs reported to modulate various biological processes and predicted to regulate as many as 30 % of human mRNAs. The miRNA targeting is determined by the nature and extent of the complementarity between an miRNA and its target sequence in the 3′UTR of mRNA. Thus, a noncoding polymorphism residing in the miRNA or the miRNA target sequence may play a role in mRNA degradation or translational repression, which post-transcriptionally regulates gene expression, with a concomitant alteration in phenotype [19, 20].

To identify miRNAs that likely target the vicinity of the rs115160714 polymorphism in the *TopBP1* 3′UTR, we utilized a computational algorithm, MicoInspector (http://mirna.imbb.forth.gr/microinspector/) that yielded three candidate miRNAs, miR-3138, miR-4302 and miR-1207-5p, whose seed sequences are complementary to the TopBP1 mRNA sequence around the rs115160714 polymorphism.

**Fig. 2:**
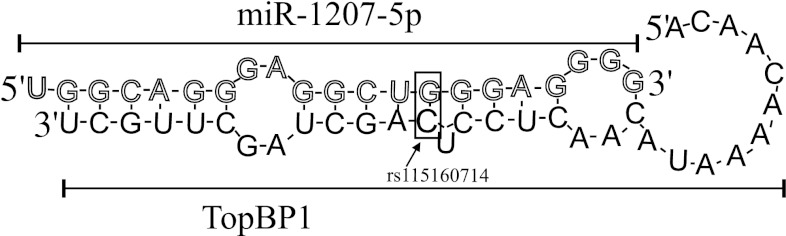
Hypothetical depicting as rs115160714 (*black* cytosine inside a *rectangle*) in 3′UTR of *TopBP1* is predicted to be targeted by miR-1207-5p. This base pairing is surrounded by the *rectangle*. miR-1207-5p sequence consists of *white letters*, while the *letters* in *TopBP1* sequence are *black*. Sequences were identified with MicroInspector algorithm (http://mirna.imbb.forth.gr/microinspector/, changed)

We did not find any literature data about the miR-3138 and miR-4302 and there is only one study concerning miR-1207-5p. Papagregoriou et al. [21] demonstrated that variant 1936T of miRSNP (rs13385) in heparin binding epidermal growth factor (HBEGF) prevents miR-1207-5p from down-regulation of HBEGF in podocytes [22]. We speculate that our findings may be explained on the basis of a similar mechanism. Figure 2 shows a hypothetical diagram of the interaction between fragment of *TopBP1* 3′UTR sequence and miR-1207-5p. Nonetheless, further experiments with other algorithms are needed to prove above speculation.

We don’t know the exact biological consequences of changes in TopBP1 expression level. However, increased expression of TopBP1 can cause deregulation of p53 activity. TopBP1 interacts with p53 binding domain and inhibits the promoter-binding activity of p53 [16]. Alteration of balance between TopBP1 and p53 activity may have impact on breast carcinogenesis.

TopBP1 is an essential protein that has numerous roles in the maintenance of genomic integrity. In particular, it is required for the activation of ATR and participates in DNA damage response and DNA replication [9]. Taking into account the role of TopBP1 in DNA damage response we were interested in exploring whether *TopBP1* SNP–breast cancer association varied according to smoking status or alcohol consumption. Tobacco smoking is the best recognized and most important risk factor of the development of malignant cancer. Tobacco smoke contains several potent chemical carcinogens and reactive oxygen species that may produce bulky adducts, oxidative DNA damage, and DNA strand breaks [23].

However, previous epidemiologic studies investigating the association of cigarette smoking and breast cancer showed inverse, null or positive associations. It has been suggested that the genetic background might modify the association between tobacco smoke and breast cancer. A few studies have shown that defective DNA repair system modestly increases tobacco-related breast cancer risk [24–28].

We found out significant breast cancer prevalence in group of smokers who were T allele carriers. Thus, polymorphisms in TopBP1 gene may modify the relationship between breast cancer and smoking.

There is strong epidemiological evidence that consumption of alcoholic beverages increases the risk of cancers of the oral cavity and pharynx, esophagus, and larynx. Alcohol drinking has also been linked to breast cancer in women [29]. Acetaldehyde, the primary metabolite of ethanol generates several types of DNA adducts that block DNA replication and affect DNA damage response [30]. Our results did not show any association between alcohol intake and increased risk of breast cancer in Polish population. *TopBP1* polymorphism did not changed alcohol consumption-breast cancer risk relationship.

In conclusion, our results showed that rs115160714 polymorphism can increase breast cancer risk and is associated with changes in TopBP1 expression.
